# Biodegradable Chitosan-Based Films as an Alternative to Plastic Packaging

**DOI:** 10.3390/foods12183519

**Published:** 2023-09-21

**Authors:** Natalia Wrońska, Nadia Katir, Marta Nowak-Lange, Abdelkrim El Kadib, Katarzyna Lisowska

**Affiliations:** 1 Department of Industrial Microbiology and Biotechnology, Faculty of Biology and Environmental Protection, University of Lodz, 12/16 Banacha Street, 90-236 Lodz, Polandkatarzyna.lisowska@biol.uni.lodz.pl (K.L.); 2 Engineering Division, Euromed Research Center, Euro-Med University of Fes (UEMF), Route de Meknes, Rond-Point de Bensouda, Fes 30070, Moroccoa.elkadib@ueuromed.org (A.E.K.)

**Keywords:** biodegradable chitosan films, packaging material, bioplastic

## Abstract

The impact of synthetic packaging on environmental pollution has been observed for years. One of the recent trends of green technology is the development of biomaterials made from food processing waste as an alternative to plastic packaging. Polymers obtained from some polysaccharides, such as chitosan, could be an excellent solution. This study investigated the biodegradability of chitosan–metal oxide films (ZnO, TiO_2_, Fe_2_O_3_) and chitosan-modified graphene films (CS-GO-Ag) in a soil environment. We have previously demonstrated that these films have excellent mechanical properties and exhibit antibacterial activity. This study aimed to examine these films’ biodegradability and the possibility of their potential use in the packaging industry. The obtained results show that soil microorganisms were able to utilize chitosan films as the source of carbon and nitrogen, thus providing essential evidence about the biodegradability of CS, CS:Zn (20:1; 10:1), and CS:Fe_2_O_3_ (20:1) films. After 6 weeks of incubation, the complete degradation of the CS-Fe_2_O_3_ 20:1 sample was noted, while after 8 weeks, CS-ZnO 20:1 and CS-ZnO 10:1 were degraded. This is a very positive result that points to the practical aspect of the biodegradability of such films in soil, where garbage is casually dumped and buried. Once selected, biodegradable films can be used as an alternative to plastic packaging, which contributes to the reduction in pollution in the environment.

## 1. Introduction

The impact of synthetic packaging on environmental pollution has been observed for years. Food packaging has undergone an unusual development because most widespread foodstuffs are being sold in plastic packaging. Nowadays, plastic is registered as the second most frequently used food packaging material [1]. This increases plastic pollution, one of the leading causes of marine animal deaths. Moreover, plastic-based packaging is often supplemented with toxic synthetic compounds (e.g., polyethylene, polypropylene, polystyrene, and other petroleum compounds) that have a negative impact on human health [2]. However, it is generally accepted that the use of long-lasting, non-biodegradable materials is inappropriate because this type of waste will remain in the environment for hundreds or even thousands of years. Polymers made from petrochemical products account for a large percentage of environmental production [3]. The WHO (World Health Organization) estimates that polluted water causes approximately 500,000 diarrheal deaths every year. Billions of tons of dumped plastic generates so-called microplastics and nanoplastics which can easily be ingested by animals, leading to a disturbance of ecosystems and causing the death of millions of children and animals every year. Moreover, due to the considerable financial outlay, the disposal of the materials is inadequate. Unfortunately, recycling policy and separate waste collection are still insufficiently implemented worldwide.

Increasing consumer demands for eco-friendly food are mobilizing the food industry to introduce biomaterial-based food packaging. Ideal food packaging material behaves as a chemical and biological barrier to preserve food quality. Its additional advantages are ease of portability and comfort of use. Moreover, it should be cheap and biodegradable, without causing the accumulation of solid waste in soil. Despite the use of bio-based resources, including polysaccharides, lipids, and proteins, as substitutes for conventional petroleum-based plastics, further investigation is needed for the rational design of ideal food packaging materials [4]. Polymers obtained from some polysaccharides, such as chitosan (CS) and cellulose, are edible and biodegradable [5]. Films made from natural polysaccharides are mostly efficient gas barriers and have moderately mechanical properties. Moreover, it has been proved that CS films can be chemically modified, blended, or reinforced with functional compounds to improve their mechanical properties, chemical and thermal stability, and even their biological responses [5,6,7,8]. Chitosan is obtained from chitin, which composes the exoskeleton of crustaceans (e.g., shrimps, crabs). It is the second most abundant polysaccharide on Earth, after cellulose. Chitosan is the deacetylated derivative of chitin and is mostly composed of (1-4)-linked 2-amino-2-deoxy-β-D-glucose monomers. The source of chitin will determine the molecular size, acetylation degree (DA), N/C ratio, and polydispersity. The degrees of acetylation (DA) for chitin and chitosan are above 90% and below 40%, respectively. These two compounds also differ in nitrogen content; for chitin, the nitrogen content is 7%, and for chitosan, it is greater than 7% [9]. The presence of amino groups in the polymer chain provides some important characteristics, e.g., the electronegative amino group takes up protons and develops a positive charge (ammonium derivative), providing chitosan with several chemical and biological properties [10]. Moreover, chitosan as a major waste product of the seafood industry generates a huge amount of crustacean shell waste. Ineffective management of this waste (it is land-filled, burned, drowned in the sea, or left to spoil naturally) leads to serious environmental hazards. These by-products should be used to produce chitin, which can be converted to chitosan using the deacetylation process. All these features make modified chitosan polymers an excellent alternative to traditional packaging. This polysaccharide can be used in the food industry in the form of flexible packaging films or coatings. Moreover, chitosan films could be modified with various compounds in order to improve their physiochemical properties. Some applications of chitosan in this area are presented in Table 1.

The replacement of synthetic polymers that are harmful to the environment with biopolymers is highly desirable and is expected to be at the heart of strategies to eradicate the threats of micro- and nanoplastics. Therefore, it seems reasonable to determine the degree of biodegradability of chitosan-based films. We previously demonstrated that the incorporation of sol–gel in metal oxides (e.g., ZnO, TiO_2_, Fe_2_O_3_, among others), or the exogenous introduction of Ag/GO through the exfoliation of graphene oxide sheets, improved the strength, flexibility, and antimicrobial properties of a set of chitosan–metal oxide films and different chitosan-modified graphene films [21,22,23,26]. This study aimed to verify these films’ biodegradability and applicability in the packaging industry.

## 2. Materials and Methods

### 2.1. Materials

Chitosan with a medium molecular weight and 85% deacetylation degree, titanium diisopropoxide bis(acetylacetanate) (Ti(acac)_2_OiPr_2_), iron (III) acetylacetonate (Fe(acac)_3_), zinc acetate (Zn(OAc)_2_), and silver nitrate (AgNO_3_) were purchased from Sigma-Aldrich (Hamburg, Germany). Graphene oxide (GO) was obtained from graphite flakes using a modified Hummers method [27].

### 2.2. Preparation of Chitosan–Metal Oxide Films

**Figure 1:**
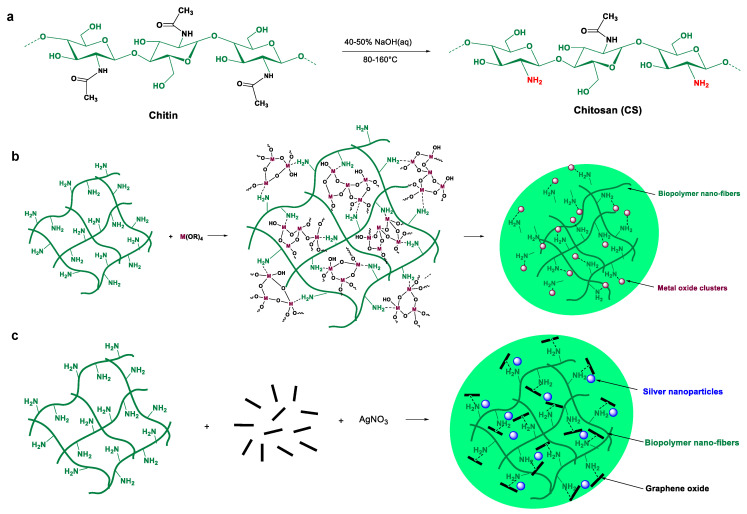
Preparation of modified chitosan films following two strategies. (**a**) Deacetylation of chitin marine waste affords chitosan. (**b**) Preparation of metal-oxide-modified chitosan using sol–gel chemistry. (**c**) Illustration of the exfoliation of graphene oxide sheets and the entrapment of silver nanoparticles inside the network.

First, 0.05 g of chitosan was dissolved in 4 mL of 1% (*v*/*v*) acetic acid solution. A given mass of the metal precursor with a NH2:M molar ratio of (1:1; 2:1; 10:1; 20:1) was added to the abovementioned solution. The mixture was then stirred for 1 h at room temperature to obtain a homogeneous dispersion, and the resulting solution was cast onto a clean Petri dish for 24 h until total evaporation of the solvent. Details and more data, e.g., from SEM, EDX, and FTIR analysis, are available in our previous work [23]. The chemical composition of CS-MOx-f is presented in Table 2. The preparation of metal (oxide)-containing chitosan films are illustrated in Figure 1.

### 2.3. Preparation of Chitosan–Graphene Film

The procedure used to prepare the CS-GO-Ag film is similar to that in a previous study [21]. Graphene oxide (GO) was obtained from graphite flakes using the modified Hummers method (82 z food spoilage). First, 1.5 mg of GO was dispersed in 2 mL of 1% (*v*/*v*) acetic acid solution and submitted to sonication. Then, 1.5 mg of AgNO_3_ was added to the GO suspension, followed by the addition of 5 mL of 1% (*v*/*v*) acetic acid solution. The mixture was stirred for one minute before the addition of 50 mg of CS. The suspension was stirred for 2 h, poured into plastic Petri dishes, and dried at RT to form films. The chemical composition of chitosan–graphene films is presented in Table 3.

### 2.4. Biodegradability of Chitosan Films

Determination of the Degradability of the CS Film using Soil Microorganisms

**Figure 2:**
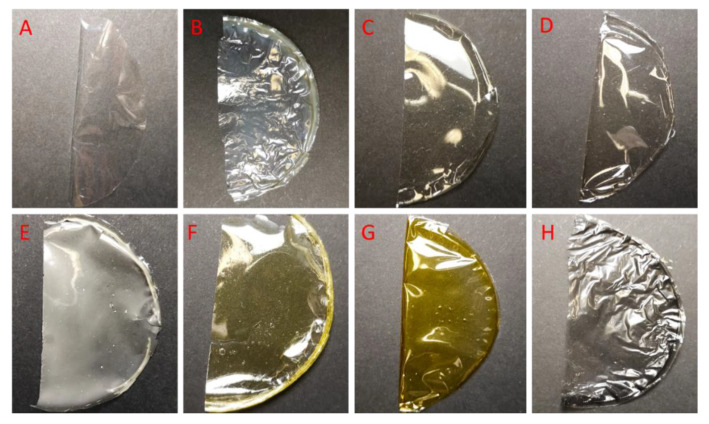
Images of the visual appearance of the chitosan, chitosan–metal and chitosan–graphene films before incubation in the soil. (**A**)—polyethylene foil, (**B**)—CS, (**C**)—CS-ZnO 2:1, (**D**)—CS-ZnO 20:1, (**E**)—CS-ZnO 10:1, (**F**)—CS-TiO_2_ 1:1, (**G**)—CS-Fe_2_O_3_ 20:1, (**H**)—CS-GO-Ag. Time incubation, t = 0.

**Figure 3:**
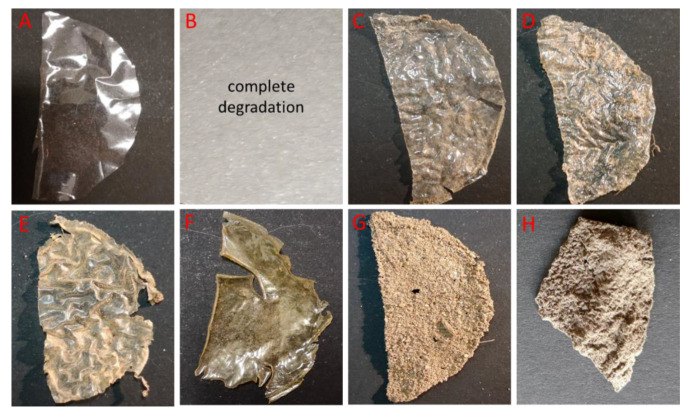
Images of the visual appearance of the chitosan, chitosan–metal and chitosan–graphene films after 4 weeks incubation in the soil. (**A**)—polyethylene foil, (**B**)—CS, (**C**)—CS-ZnO 2:1, (**D**)—CS-ZnO 20:1, (**E**)—CS-ZnO 10:1, (**F**)—CS-TiO_2_ 1:1, (**G**)—CS-Fe_2_O_3_ 20:1, (**H**)—CS-GO-Ag.

**Figure 4:**
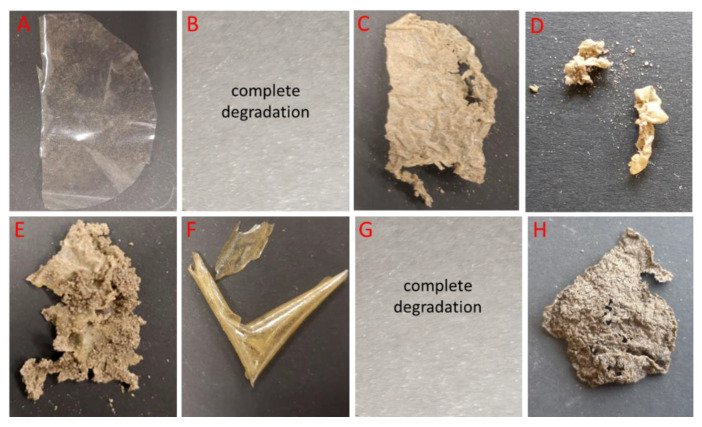
Images of the visual appearance of the chitosan, chitosan–metal and chitosan–graphene films after 6 weeks incubation in the soil. (**A**)—polyethylene foil, (**B**)—CS, (**C**)—CS-ZnO 2:1, (**D**)—CS-ZnO 20:1, (**E**)—CS-ZnO 10:1, (**F**)—CS-TiO_2_ 1:1, (**G**)—CS-Fe_2_O_3_ 20:1, (**H**)—CS-GO-Ag.

**Figure 5:**
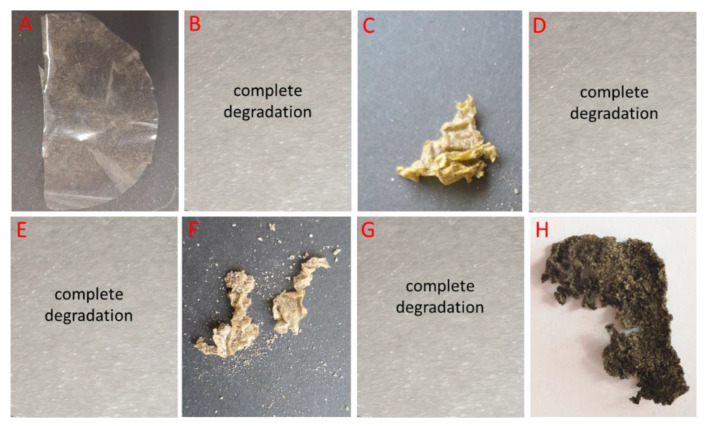
Images of the visual appearance of the chitosan, chitosan–metal and chitosan–graphene films after 8 weeks incubation in the soil. (**A**)—polyethylene foil, (**B**)—CS, (**C**)—CS-ZnO 2:1, (**D**)—CS-ZnO 20:1, (**E**)—CS-ZnO 10:1, (**F**)—CS-TiO_2_ 1:1, (**G**)—CS-Fe_2_O_3_ 20:1, (**H**)—CS-GO-Ag.

The study presents an assessment of the biodegradability of chitosan films via the determination of the degradability of the CS films by soil microorganisms. The test was performed on a laboratory scale following a method described by Seoane et al., (2017), with some modifications [28]. The soil samples were collected in the garden of the Faculty of Biology and Environmental Protection University of Lodz, Poland, and used as an incubation medium. The soil was maintained at room temperature, with 45% moisture, and pH equal to 6. The soil was placed in a special container and sterile samples were buried at a depth of approximately 10 cm (each sample in a separate container) and incubated at room temperature. The buried samples were dug out at different periods of time (4, 6, and 8 weeks of incubation) and rubbed gently with a brush to remove soil residues. Then, the samples were rinsed thoroughly with distilled water, dried at 50 °C for 4 h, desiccated for 24 h at room temperature, weighed, and the percentage of weight loss of the samples was determined. We analyzed 8 films: A—polyethylene foil, B—CS, C—CS-ZnO 2:1, D—CS-ZnO 20:1, E—CS-ZnO 10:1, F—CS-TiO_2_ 1:1, G—CS-Fe_2_O_3_ 20:1, H—CS-GO-Ag. Polyethylene foil was used as a negative control. The obtained images of CS films were compared with images after 4-, 6-, or 8-week periods of incubation. The results are shown in Figure 2, Figure 3, Figure 4 and Figure 5.

### 2.5. Solubility in Water

The water solubility of selected films was estimated gravimetrically according to the method described by Kaya et al., (2018) [29]. The film sample was cut into 2 × 3 cm^2^ pieces. Then, the initial dry weights were estimated for all tested samples. Next, chitosan modified films were incubated for 24 h at 24 °C in Petri dishes containing 30 mL of deionized water. After incubation, each sample was dried at 50 °C for 24 h. The tested samples were weighed and the weight loss % was calculated according to the following equation.

%WL = weight loss/initial weight × 100

## 3. Results and Discussion

### 3.1. Preparation of Metal (Oxide)-Containing Chitosan Films

The two approaches used for the preparation of metal (oxide)-containing materials are illustrated in the following figures (Figure 1). For the sol–gel modification, a suitable amount of the metal alkoxide precursor is introduced in a colloidal chitosan solution. The latter behaves as a template for controlling the growth of the metal oxide during the steps of hydrolysis and condensation. The evaporation of the film induces further condensation and results in shaping flexible chitosan films entrapping metal oxide nanoparticles. Notably, the amount of the starting metal alkoxide with respect to chitosan resulted in a well-defined molar ratio of chitosan: metal oxide. Consequently, films with increasing metal loading (e.g., zinc oxide) could be obtained and assessed in terms of biodegradability.

The second approach deals with the incorporation of Ag/GO as a ternary component. Starting from 3 wt% of graphene oxide, we previously demonstrated that such loading could be exfoliated by chitosan, with the simultaneous entrapment of silver nanoparticles. After solvent evaporation, functional chitosan films with flexibility could be obtained. It should be mentioned that the incorporation of silver was dictated by its outstanding antibacterial and antiviral properties, making it the most powerful chemical species against microbes and viruses.

A detailed characterization can be found in our previous reports [21,22,23]. The in situ modification of chitosan in liquid phase synthesis using metal alkoxide was found to be more convenient compared to the mechanical dispersion of a metal oxide already inside the chitosan network.

### 3.2. Biodegradation of Chitosan Films in Soil

The chitosan films were selected for the biodegradability test due to their good mechanical, thermal, and antibacterial properties [21,22,23].

All tested films were homogeneous and were stored at room temperature. Each film was halved and photographed before being placed in the soil (Figure 2).

The biodegradation of these films in a soil environment was investigated and compared with that of pure chitosan films. The negative control was a commercial, non-biodegradable polyethylene film. The results were documented with photos, which are shown in Figure 3, Figure 4 and Figure 5. In soil placed in separate containers and incubated at room temperature, all pure chitosan films were degraded. Our further experiments showed that the pure chitosan film was biodegradable after just 2 weeks. The other films were wrinkled and pliable. There was a slight loss of chitosan films with the addition of titanium and zinc (Figure 3). Our results clearly show that chitosan modifications can significantly affect the decomposition of tested materials.

When chitosan films are subjected to biodegradation, a complex series of processes unfolds, leading to the breakdown of the polymer chains and the eventual transformation of these films into simpler compounds. At the onset of biodegradation, water permeates the chitosan film, initiating a swelling process. This hydration step promotes the diffusion of enzymes and microorganisms into the film matrix. Enzymatic degradation is one of the primary mechanisms responsible for the breakdown of chitosan films. Several enzymes, including chitosanase, lysozyme, and proteases, act upon the glycosidic bonds present in chitosan, hydrolyzing them into smaller fragments. The initial stage of the proposed chitosan degradation mechanism is depolymerization (random splitting of β-1,4-glycosidic bonds) and subsequent deacetylation (hydrolysis of N-acetyl linkage). As a consequence, an increase in the degree of deacetylation and a decrease in molecular weight are observed [30]. Cleavage of the chitosan functional groups (amino, carbonyl, amide, and hydroxyl) can occur at the same time depending on the chemical and/or enzymatic conditions. Chitosanase, specifically, plays a crucial role in the initial stages of degradation, cleaving the chitosan chains at random points [31]. This enzymatic hydrolysis process gradually breaks down the chitosan film into oligosaccharides and eventually into individual monomers, such as glucosamine (D-glucosamine or N-acetyl-glucosamine), which can be further utilized by microorganisms [30].

As expected, no change was observed in the negative control, polyethylene (PE) film, after 8 weeks of incubation in a soil environment at room temperature (Figure 5).

Macroscopic modifications in the tested chitosan films, such as the roughening of their surface and formation of holes and cracks, are indications that biodegradation is occurring [32]. The obtained results show that soil microorganisms were able to utilize chitosan films as a source of carbon and nitrogen, thus providing significant evidence for the biodegradability of CS, CS:Zn (20:1; 10:1), and CS:Fe_2_O_3_ (20:1) films. Pure chitosan films were utilized the fastest. After 6 weeks of incubation, the complete degradation of the CS-Fe_2_O_3_ 20:1 sample was noted (Figure 4), while after 8 weeks, CS-ZnO 20:1 and CS-ZnO 10:1 (Figure 5) were degraded. This is a very positive result that points to a practical aspect of the biodegradability of such films in soil, where garbage is casually dumped and buried. In the case of the other tested films, the biodegradation process was slower. It was also observed that the film with the addition of graphene and silver was the slowest to degrade in the soil environment. This draws particular attention due to the already high presence of silver in the environment [33]. Some data suggest that graphene can degrade and transform in a water environment via the action of enzymes and the Fenton reaction, resulting in some graphene mineralization (CO_2_) and the generation of more oxidized graphene species [34]. There are very little data showing the degradation of graphene oxide via soil microorganisms. Navarro et al. (2020) assessed the potential mineralization and release of GO in a soil environment. The results show that the conversion of GO to CO_2_ was negligible (<2%) in soil. The obtained data suggest the high bio(degradation) stability of GO, which is likely due to its limited availability resulting from its rapid homo/hetero-aggregation with soil colloids [35]. Some mineralization (9%) of graphene accumulated by hydroponically grown rice plants has been observed [36]. In the case of graphene-based chitosan materials, which due to their properties are promising materials for many industries, it should be remembered that the fate of these materials in the environment should be thoroughly investigated.

**Figure 6:**
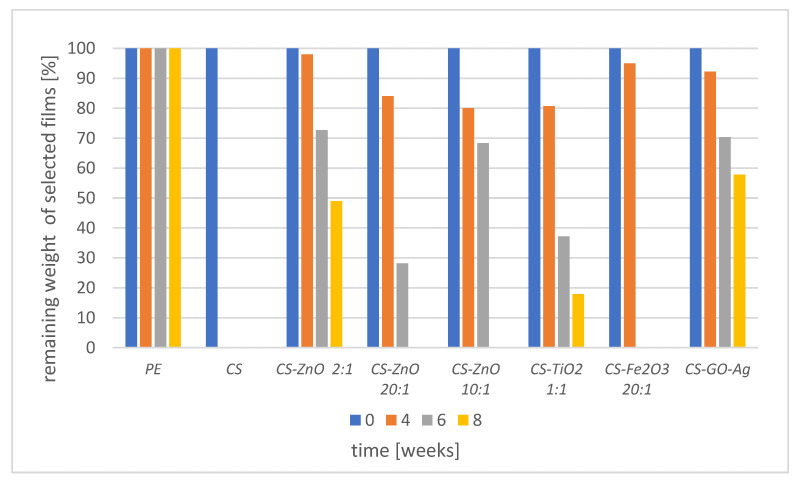
Change in the weight ratio in (PE) polyethylene foil, CS, CS-ZnO 2:1, CS-ZnO 20:1, CS-ZnO 10:1, CS-TiO_2_ 1:1, CS-Fe_2_O_3_ 20:1, CS-GO-Ag films exposed to soil environment after 4, 6 and 8 weeks of incubation time.

Moreover, the extent of weight loss of tested materials was studied. The results are presented in Figure 6.

Figure 6 shows the change in the weight ratio of tested samples in 4, 6, and 8 weeks of incubation time. No change was observed in the weight of the PE film after 8 weeks of incubation. In the case of films where zinc was the metal precursor, the weight losses (incubation time—6 weeks) in CS-ZnO 2:1, CS-ZnO 10:1, and CS-ZnO 20:1 were 27,3%, 31.7%, and 71.8% respectively. This may be related to the chitosan content in these samples. In the film with a higher content of CS, the weight loss was faster. Kuo et al. (2006) had similar observations while investigating a chitosan/nylon blending film [37]. After 8 weeks of incubation of CS-ZnO 10:1 and CS-ZnO 20:1, their complete degradation was noted. The complete degradation of these films was possible due to the enzymes of soil microorganisms [38]. It was also indicated that the diffusion of water into the polymer matrix may cause swelling and accelerate the biodegradation process of the films [39]. The weight loss was gradual in the CS-TiO_2_ 1:1 film and amounted to almost 20% at 8 weeks of incubation time. A similar effect was noted in the case of the CS-GO-Ag film, but the weight loss was much slower.

**Figure 7:**
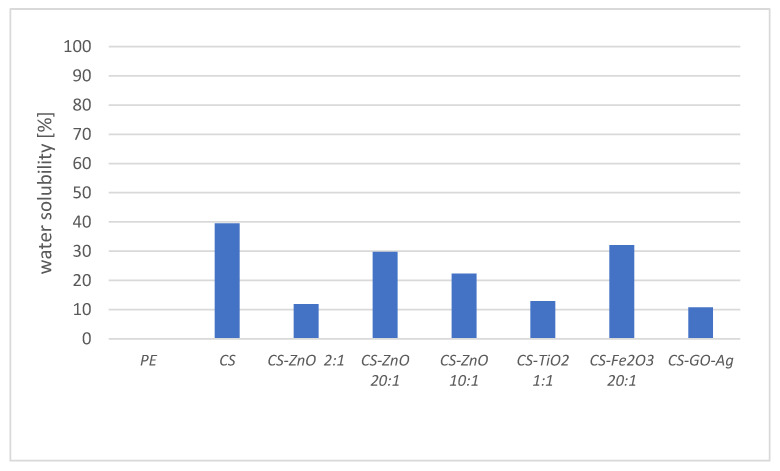
Water solubility of the chitosan, chitosan–metal and chitosan–graphene films after 24 h incubation in the deionized water.

On the one hand, polymer-based films must be easily biodegradable, but on the other hand, they must have a certain stability for packaging food throughout its shelf life. Films with poor water resistance may soften in high humidity conditions, and thus the molecules can pass through the film more easily as a result of changes in the film structure [40]. We observed that pure chitosan film was the most water-soluble (water solubility rate was recorded as 39.5%). Our data (39.5%) were lower than the values reported by Chang et al. (2021) and Akyuz et al. (2018): 45.7% and 58%, respectively. On the other hand, our score was higher than the results (24%) of Kaya et al. (2018) [29]. With the addition of metal oxide or graphene to the chitosan film matrix, the water solubility of the composites decreased (Figure 7). This may be due to intermolecular interactions between chitosan and metal oxides or graphene. Moreover, the addition of these components reduces the hydrophilicity of the tested films.

There are different types of microorganisms in the soil. Depending on the growth temperature, some are thermophilic, while others are psychrophilic or mesophilic. According to the change in temperature, the biological activity in soil shifts from one to another group. The soil and the microorganisms inhabiting it are heterogeneous, and obtaining uniform samples is practically impossible; therefore, describing ecological relationships is difficult [41]. Chitosanases, which catalyze the hydrolytic degradation of chitosan, are widely found in soil microorganisms and some plants. Several species of chitosanases producing microorganisms have been confirmed, e.g., *Bacillus*, *Arthrobacter*, *Penicillium*, *Aspergillus*, and *Streptomyces* [42,43].

In our research, we showed that the unmodified chitosan film (CS) was decomposed most quickly in the soil environment (after 2 weeks). Studies have shown that CS can be biodegraded to non-toxic residues [44]. The rate of its degradation may depend largely on the molecular mass and its deacetylation degree [45]. The relationship between chitosan biodegradability and degree of deacetylation is also dependent on crystallinity. The decrease in chitosan crystallinity results in an increase in the degradation rate of this polymer. The crystallinity of chitosan, and thus its degradation, is also influenced by acetyl residues located along its chain. It is known that chitosan with a lower molecular weight is degraded faster [30,46]. Nakashima et al. (2006) also tested the biodegradability of chitosan films via indoor and outdoor tests. The weight loss values of chitosan films were 31.7, 77.6, and 100% after cultivating with *Sphingobacterium multivorum* for 0.5, 1, and 1.5 months, respectively [47]. They obtained similar results in the outdoor test when the experiment was carried out using chitosan-degrading bacteria from the soil. Other studies showed the biodegradation of a polyethylene–chitosan (PE-chitosan) film in a soil environment. In soil stored in the laboratory conditions, 73.4% of the chitosan was degraded after 6 months of incubation. The same CS film, buried in an open field, was 100% degraded. It was shown that the PE-chitosan film degraded faster than the commercially available starch-based film [48]. Pavoni et al., (2019) observed the beginning of biodegradation of the cornstarch/chitosan-based films after 15 days of analysis [49]. Kuo et al. (2006) showed that the percentage of chitosan addition significantly affects the degree of biodegradability of nylon 11/chitosan blend films. In the blending film made with 50% chitosan and nylon 11, the percentage of weight loss is four times more than that in pure nylon 11. They also observed more cavities in the tested films when chitosan supplementation increased.

Many studies report that chitosan coatings are eco-friendly, easily biodegradable, and in most cases, edible [9,29]. Other researchers report that incorporating chitosan into synthetic polymers can significantly improve the rate of degradation of plastics [50]. The most commonly used synthetic polymer for chitosan is polyvinyl alcohol (PVA), which is water-soluble and non-toxic.

## 4. Conclusions

Currently, the packaging industry relies heavily on the use of petroleum-based synthetic plastic materials. Unfortunately, the non-degradable nature of these plastics leads to serious difficulties with municipal waste disposal. For this reason, it is necessary to look for alternative, biodegradable packaging that would help solve the problem of environmental pollution. The results show that soil microorganisms were able to utilize chitosan films as a source of carbon and nitrogen, thus providing significant evidence for the biodegradability of CS, CS:Zn (20:1; 10:1), and CS:Fe_2_O_3_ (20:1) films. After 6 weeks of incubation, complete degradation of the CS-Fe_2_O_3_ 20:1 sample was noted, while after 8 weeks, CS-ZnO 20:1 and CS-ZnO 10:1 were degraded. The obtained data suggest the possibility of using composite films as a green and eco-friendly approach for packaging food products, replacing petroleum-based synthetic plastics. We also call for precautions when graphene derivatives are used, as a slow rate of biodegradability was noticed for chitosan films incorporating graphene oxide. In this case, graphene should be combined with chemicals that could trigger its fast decomposition. On the whole, these investigations provide additional guidelines toward the rational design of new environmentally friendly packaging material, since the resulting CS films displayed good biodegradability.

## Figures and Tables

**Table 1:** Application of CS in food packaging.

Type of Chitosan Film	Properties	References
CS/ZnO	improvement of the quality of pork meat during cold storage	[11]
(CS)/Fe_3_O_4_, (Gr)/CS/Fe_3_O_4_	antimicrobial activity	[12]
CS/GEL/AgNPs	antimicrobial activity	[13]
CS/TiO_2_ NPs	exposure to UV light, triggered higher antimicrobial activity	[14]
CS/TiO_2_ NPs	antimicrobial, antioxidant, and ethylene scavenging properties	[15]
CS/TiO_2_ NPs	good antimicrobial activity under dark and light conditions	[16]
RS/CMCh	enhanced antioxidant and antimicrobial properties	[17]
CS/garlic essential oil	antioxidant and antimicrobial activity	[18]
CS	antioxidant system of figs during storage	[19]
CS/propolis coatings	antioxidant system of strawberries during storage	[20]
CS/GO films	antimicrobial activity	[21]
CS/metal oxide films CS/GO films	antimicrobial activity	[22,23]
DPPS-CH (chitosan bearing pyrazole derivative)	antimicrobial activity	[24]
Cs-EATT Cs-BATT (chitosan bearing 1,3,4-thiadiazole derivative)	antimicrobial activity	[25]

**Table 2:** Chemical composition of CS-MOx-f.

Sample Code	Metal Precursors	Molar Ratio NH_2_: Metal Precursor
PE	-	
CS	-	
CS-ZnO 2:1	Zinc acetate	2:1
CS-ZnO 10:1	Zinc acetate	10:1
CS-ZnO 20:1	Zinc acetate	20:1
CS-TiO_2_ 1:1	Titanium diisopropoxide bis(acac)	1:1
CS-Fe_2_O_3_ 20:1	Iron(III) acetylacetonate	20:1

**Table 3:** Chemical composition of chitosan–graphene films.

Sample Code	Functionalized Graphene Fillers	Metal Precursors
CS-GO-Ag	GO (3 wt%)	Silver nitrate (3%)

## Data Availability

The data presented in this study are available on request from the corresponding author.
